# Association of Immunoglobulin Levels, Infectious Risk, and Mortality With Rituximab and Hypogammaglobulinemia

**DOI:** 10.1001/jamanetworkopen.2018.4169

**Published:** 2018-11-02

**Authors:** Sara Barmettler, Mei-Sing Ong, Jocelyn R. Farmer, Hyon Choi, Jolan Walter

**Affiliations:** 1 Allergy and Clinical Immunology Unit, Division of Rheumatology, Allergy & Immunology, Massachusetts General Hospital, Boston; 2 Department of Population Medicine, Harvard Medical School, Harvard Pilgrim Health Care, Boston, Massachusetts; 3 Rheumatology Unit, Division of Rheumatology, Allergy & Immunology, Massachusetts General Hospital, Boston; 4 Division of Allergy & Immunology, Department of Pediatrics, Massachusetts General Hospital, Boston; 5 Division of Pediatric Allergy/Immunology, Department of Pediatrics, University of South Florida, Johns Hopkins All Children’s Hospital, St Petersburg

## Abstract

**Importance:**

Rituximab is an anti-CD20 chimeric antibody used in a wide variety of clinical indications. There has not been widespread adoption of consistent immune monitoring before and after rituximab therapy. However, there is a subset of patients who develop prolonged, symptomatic hypogammaglobulinemia following rituximab, and monitoring before and after rituximab therapy could help to identify these patients and initiate measures to prevent excess morbidity and mortality.

**Objective:**

To determine the current levels of screening for hypogammaglobulinemia (specifically, low immunoglobulin G), infectious risks associated with hypogammaglobulinemia, and variables associated with an increased risk of mortality.

**Design, Setting, and Participants:**

A cohort study was conducted of 8633 patients receiving rituximab from January 1, 1997, to December 31, 2017, at a large, tertiary referral center (Partners HealthCare System).

**Exposures:**

Rituximab administration.

**Main Outcomes and Measures:**

The primary outcome measures were immunoglobulin measurements, infectious complications, and mortality. Cox regression analysis was used to examine the results of infectious complications on survival, adjusted for age, sex, and indication for rituximab use.

**Results:**

Of the 8633 patients who received rituximab in the large, academic, health care system, 4479 satisfied inclusion criteria, with a mean (SD) age of 59.8 (16.2) years; 2280 patients (50.9%) were women. Most patients (3824 [85.4%]) did not have immunoglobulin levels checked before rituximab therapy. Of those who had levels determined, hypogammaglobulinemia was noted in 313 (47.8%) patients before initiation of rituximab. Following rituximab administration, worsening hypogammaglobulinemia was noted. There was an increase in severe infections after rituximab use in the study cohort (from 17.2% to 21.7%; *P* < .001). In the survival analysis, increased mortality was associated with increasing age (hazard ratio [HR], 1.02; 95% CI, 1.01-1.02; *P* < .001), male sex (HR, 1.14; 95% CI, 1.02-1.28; *P* = .02), and severe infectious complications in the 6 months before (HR, 3.14; 95% CI, 2.77-3.55; *P* < .001) and after (HR, 4.97; 95% CI, 4.41-5.60; *P* < .001) the first rituximab infusion. A total of 201 patients (4.5%) received immunoglobulin replacement following rituximab, and among these patients, higher cumulative immunoglobulin replacement dose was associated with a reduced risk of serious infectious complications (HR, 0.98; 95% CI, 0.96-0.99; *P* = .002).

**Conclusions and Relevance:**

Many patients are not being screened or properly identified as having hypogammaglobulinemia both before and after rituximab administration. Monitoring of immunoglobulin levels both before and after rituximab therapy may allow for earlier identification of risk for developing significant infection and identify patients who may benefit from immunoglobulin replacement, which may in turn help to avoid excess morbidity and mortality.

## Key Points

**Question:**

In patients receiving rituximab, what are the current rates of screening and recognition of hypogammaglobulinemia, and what are the infectious risks and predictors for increased mortality?

**Findings:**

In a cohort study of 4479 patients receiving rituximab, many patients were found as not being screened or not being properly identified as having hypogammaglobulinemia. Following rituximab therapy, there was a significant increase in severe infections in the overall study cohort, increased mortality was associated with severe infections in the 6 months before and after rituximab therapy, and higher cumulative doses of immunoglobulin replacement therapy were associated with a reduced risk of severe infections.

**Meaning:**

Many patients are not being screened or properly identified as having hypogammaglobulinemia before or after rituximab therapy, which may contribute to inferior outcomes with excess morbidity and mortality; monitoring routine serum immunoglobulin levels before and after rituximab therapy may help identify patients at high risk for developing infections and who may benefit from immunoglobulin replacement therapy.

## Teaser

This cohort study examines the association of screening for hypogammaglobulinemia with the risk of infection and mortality in patients who received rituximab therapy.

## Introduction

Rituximab is an anti-CD20 chimeric monoclonal antibody that depletes CD20-expressing B cells. Rituximab is used in a broad range of conditions, including cancer, rheumatologic conditions, and primary immunodeficiency. Current US Food and Drug Administration–approved indications include non-Hodgkin lymphoma, chronic lymphocytic leukemia, rheumatoid arthritis, granulomatosis with polyangiitis, and microscopic polyangiitis.^1^ In addition, there are many off-label uses in other specialties^2^ and expanding indications for rituximab in common variable immunodeficiency (CVID)^3^ and ongoing combination therapy trials.^4^

The mechanism of action for rituximab is thought to be cell death via direct cytotoxicity, complement-dependent cytotoxicity, and antibody-dependent cellular cytotoxicity^5,6^ and, given these effects, peripheral B-cell depletion is expected. The duration of B-cell depletion was initially estimated to be 6 to 9 months with rituximab treatment alone^7^ and 18 to 24 months in patients who received a combination of chemotherapy and rituximab.^7^ However, following these initial studies, a number of publications have described prolonged hypogammaglobulinemia (particularly low immunoglobulin G [IgG] levels) in a subset of patients receiving rituximab.^8,9,10,11,12,13^ Although initial rituximab studies did not show significant increases in infection rate,^14^ there have been reports notable for serious infection rates of 4.31 per 100 patient-years in 1 study,^15^ and 5.2 per 100 patient-years in the rituximab group compared with 3.7 per 100 patient-years in the placebo group in another study,^16^ in addition to reports of patients with prolonged, symptomatic hypogammaglobulinemia requiring immunoglobulin replacement (IgR).^8,17^

There have been some advances toward routine immunologic screening in certain subsets of patients and subspecialties given these findings. The Rituximab Consensus Expert Committee recommended that before initiating rituximab for rheumatoid arthritis treatment, a baseline IgG level should be obtained, in addition to measuring IgG levels before each rituximab cycle and longitudinally.^18^ Buch et al^18^ reported that, in both the registry data and clinical trial–compiled data,^19^ low levels of IgG prior to rituximab were associated with an increased risk of serious infections. Sustained low IgG levels (≥4 months) were associated with increased risk for serious infections in open extension studies.^19^ Despite these recommendations, the widespread adoption of a basic immunologic workup, including regularly checking immunoglobulin levels before and after the initiation of rituximab, has not occurred across the many specialties in which rituximab treatment is indicated.

Given that many patients do not undergo immunologic evaluation before rituximab treatment, it can be difficult to determine whether underlying immune dysfunction was present at baseline (eg, primary immunodeficiency, such as CVID) or if the dysfunction is a long-lasting effect (secondary immunodeficiency).^8^ Common variable immunodeficiency is a heterogeneous collection of syndromes characterized by impaired B-cell differentiation, defective immunoglobulin production, and recurrent infections. In addition to recurrent sinopulmonary infections, patients with CVID are at increased risk for autoimmunity, granulomatous disease, and cancer,^20,21,22,23,24^ which may be treated with rituximab^2,3,25^ and can present up to decades before CVID is diagnosed.^21,26^ Thus, evaluating pretreatment immunoglobulin levels may help to identify patients with underlying immunodeficiency and/or humoral dysfunction. Monitoring immunoglobulin levels following rituximab therapy may help to identify patients at risk for severe infections secondary to hypogammaglobulinemia in whom the initiation of IgR may be warranted. Memory B cells have been suggested as a marker of rituximab response, need for IgR, and recovery of cell counts/immunoglobulins.^27^

We hypothesize that in hypogammaglobulinemia after rituximab, there may be 2 subsets of patients: 1 group with normal recovery of immunoglobulin levels/cell counts and another group with long-lasting, symptomatic, hypogammaglobulinemia. In patients with persistent hypogammaglobulinemia, there is likely an increased risk for infection given the humoral dysfunction/deficiency, and these patients may benefit from IgR. The aim of our study was to evaluate the outcomes of patients who received rituximab to improve clinical standards for monitoring across specialties.

## Methods

### Study Design and Cohort

We analyzed a retrospective cohort of patients who received rituximab at our large, academic tertiary care center (Partners HealthCare System), which comprises Massachusetts General Hospital and Brigham and Women’s Hospital. We identified 8633 patients who received rituximab between January 1, 1997, and December 31, 2017, captured in the Research Patient Database Registry. The data were deidentified for analysis. Institutional review board approval with waiver of informed consent was obtained from Partners HealthCare System for this retrospective analysis. This study followed the Strengthening the Reporting of Observational Studies in Epidemiology (STROBE) reporting guideline.

To define a cohort of patients with adequate follow-up and avoid confounding by patients who were not regularly followed up at these institutions and may have had rituximab exposure at an outside institution, we confined the study cohort to those who visited the hospital at least once every 6 months in the year before the first recorded rituximab use. We further restricted the study cohort to patients who had adequate follow-up visits during the study period, defined as having at least 1 visit every 6 months in the 18 months following rituximab use (ie, ≥1 visit within the first 6 months, ≥1 visit between 6 and 12 months, and ≥1 visit between 12 and 18 months of rituximab use). Patients who died during the follow-up period and therefore did not have follow-up visits throughout the follow-up period were included in the study cohort. Patients younger than 14 years were excluded.

We evaluated patient demographics, including age, sex, race/ethnicity, and vital status. The indication for rituximab was evaluated by *International Classification of Diseases, Ninth Revision* (*ICD-9*) and *International Statistical Classification of Diseases, 10th Revision* (*ICD-10*) major diagnostic groups. These subgroups included lymphoma/malignancy (including non-Hodgkin lymphoma and chronic lymphocytic leukemia), autoimmune/rheumatologic diseases (including rheumatoid arthritis, systemic lupus erythematosus, antineutrophil cytoplasmic antibody–associated vasculitis, granulomatosis with polyangiitis, and microscopic polyangiitis), hematologic conditions (including idiopathic thrombocytopenic purpura, thrombotic thrombocytopenic purpura, autoimmune hemolytic anemia), and primary immunodeficiencies (specifically CVID).

### Immunologic Evaluation for Hypogammaglobulinemia

The records were queried for immunoglobulin levels, particularly IgG, to evaluate how many patients had immunoglobulin levels checked and if patients had hypogammaglobulinemia in the 12 months before and 18 months following rituximab administration. Hypogammaglobulinemia was defined as a serum IgG level below 600 mg/dL and further stratified into mild, 400 to 599 mg/dL; moderate, 200 to 399 mg/dL; and severe, 0 to 199 mg/dL (to convert to grams per liter, multiply by 0.01). In this analysis, we included only patients older than 14 years to avoid confounding by age-adjusted immunoglobulin ranges (excluding 52 pediatric patients). We further examined if patients with a low IgG level had a clinical diagnosis of hypogammaglobulinemia documented in the medical record.

### Infectious Complications

To examine the prevalence of severe infections among patients treated with rituximab, we quantified the number of patients who had 1 or more severe infections before and after the index rituximab infusion. Severe infections were defined as infections requiring hospitalizations and were assessed at certain time points to account for the long duration of action of rituximab, specifically, at 6 and 12 months before and 6, 12, and 18 months following the initiation of rituximab therapy, with adjustment for patients who died during the 18-month follow-up period. The analysis was stratified by disease groups (ie, cancer, rheumatologic disease, CVID, and hematologic disease). We further compared the prevalence of serious infections among patients who had IgG levels within the reference range and those with hypogammaglobulinemia prior to rituximab use.

### B-Cell Phenotyping

We evaluated flow cytometry data to determine the use of B-cell phenotyping, including switched-memory B cells, to see if there was a correlation between switched-memory B cells and the patients who had prolonged hypogammaglobulinemia and required IgR. Prolonged hypogammaglobulinemia was considered a duration of B-cell depletion beyond the initial estimations of 6 to 9 months in rituximab treatment alone and 18 to 24 months in a combination of chemotherapy and rituximab.

### Survival Analysis

We performed Cox regression analysis to examine the association between infectious complications and survival. The outcome of interest was survival time since initiation of rituximab therapy. Patients were followed up until death or were censored at the end of the follow-up period, which was defined as 18 months after rituximab use. The primary covariate of interest was the occurrence of serious infectious complications within 6 months following the first rituximab infusion, expressed as a time-varying covariate. The models were adjusted for age, sex, the indication for rituximab use (ie, cancer, rheumatologic disease, hematologic disease, CVID), and the occurrence of serious infectious complications 6 months prior to the first rituximab use. Patient age was expressed as a continuous variable, and all other covariates were expressed as binary variables.

In addition, subgroup analyses were performed to evaluate the associations between serious infectious complications and survival for each disease subgroup. We hypothesized that serious infectious complications both before and after rituximab use are significant predictors of survival among patients who received rituximab therapy. We chose to examine serious infectious complications in the 6 months before and after rituximab initiation since our data showed that infectious rates were highest during these periods and a prior study reported that 79% of serious infections that occurred following rituximab use developed within the first 6 months of treatment.^19^

### IgR and Treatment Effects

We performed Cox regression analysis to examine the treatment effect of IgR on the occurrence of serious infectious complications following rituximab use. The outcome of interest was time to the occurrence of a serious infectious complication requiring hospitalization since initiation of rituximab; patients were censored at 18 months following rituximab use. The primary covariate of interest was IgR treatment dose since the first rituximab infusion, expressed as a time-varying covariate (in grams). Given that important differences in baseline characteristics likely exist between patients who received IgR and those who did not (eg, greater risk for or higher numbers of infections may have prompted the initiation of IgR in some patients), direct comparison between the 2 groups would likely yield an inaccurate assessment of the outcome of IgR. We therefore developed 2 Cox regression models: the first included all patients and the second considered only those who received IgR. All models were adjusted for age, sex, the indication for rituximab use (ie, cancer, rheumatologic disease, hematologic disease, CVID), and the occurrence of serious infectious complications 6 months before the first rituximab use.

### Statistical Analysis

Descriptive statistics were applied to examine trends in severe infections before and after the initiation of rituximab, and *z* statistics were applied to compare the proportion of patients who had a serious infection at 2 time points: in the 6 months before and the 6 months after the index rituximab infusion. All tests of statistical significance were 2-tailed with an α level of *P* < .05. Statistical analysis was conducted with R, version 3.3.0 (R Foundation).

## Results

### Demographics and Indication for Rituximab Use

We identified a total of 8633 patients who received rituximab in the Partners HealthCare System from 1997 to 2017. Of these, 4479 satisfied inclusion criteria for adequate follow-up before and after rituximab therapy and were 14 years or older (eFigure in the Supplement). There were 2280 women (50.9%) and 2198 men (49.1%); sex was not documented for 1 patient. The mean (SD) and median ages were 59.8 (16.2) and 61 years, respectively. Most patients were non-Hispanic white (3807 [85.0%]) (Table 1). The median number of visits every 6 months before and after rituximab initiation ranged between 5 and 16. The eTable in the Supplement provides a detailed description of the distribution of follow-up visits. The indication for rituximab by *ICD-9 *or *ICD-10* major diagnostic groups was cancer in 3478 patients (77.7%), autoimmune disorder in 1241 patients (27.7%), hematologic disorder in 340 patients (7.6%), and primary immunodeficiency or CVID in 57 patients (1.3%). There was overlap between these groups, with some patients having concomitant diagnoses in multiple major diagnostic groups (Table 1).

**Table 1.** Demographics of Patients Receiving Rituximab at the Partners Healthcare System Between 1997 and 2017 (N = 4479)

Characteristic	No. (%)
Sex	
Men	2198 (49.1)
Women	2280 (50.9)
Race/ethnicity	
American Indian	6 (0.1)
Asian	90 (2.0)
Black/African American	217 (4.8)
Native Hawaiian	2 (0.04)
Non-Hispanic white	3807 (85.0)
Hispanic	166 (3.7)
Other/unknown	357 (8.0)
Age, quartile, y	
1st	50.0
2nd, median	61.0
3rd, mean (SD)	59.8 (16.2)
4th	72.0
Age, y	
Median	61
Mean (SD)	59.8 (16.2)
Indications for rituximab use	
Cancer	3478 (77.7)
Rheumatologic disorder	1241 (27.7)
CVID	57 (1.3)
Hematologic disorder	340 (7.6)
Died	1248 (27.9)
Cancer (n = 3478)	1130 (32.5)
Rheumatologic disorder (n = 1241)	166 (13.4)
CVID (n = 57)	6 (10.5)
Hematologic disorder (n = 340)	93 (27.4)
Immunoglobulin levels before initiation of rituximab therapy	
IgG within reference range	342 (7.6)
Hypogammaglobulinemia^a^	
Mild	91 (2.0)
Moderate	65 (1.5)
Severe	157 (3.5)
Not checked	3824 (85.4)
Immunoglobulin levels after initiation of rituximab therapy	
IgG within reference range	195 (4.4)
Hypogammaglobulinemia^a^	
Mild	112 (2.5)
Moderate	102 (2.3)
Severe	149 (3.3)
Not checked	3921 (87.5)
Moderate to severe hypogammaglobulinemia before initiation of rituximab therapy^a^	
Cancer (n = 3478)	186 (5.3)
Rheumatologic disorder (n = 1241)	70 (5.6)
CVID (n = 57)	11 (19.3)
Hematologic disorder (n = 340)	26 (7.6)
Moderate to severe hypogammaglobulinemia after initiation of rituximab therapy^a^	
Cancer (n = 3478)	216 (6.2)
Rheumatologic disorder (n = 1241)	68 (5.5)
CVID (n = 57)	6 (10.5)
Hematologic disorder (n = 340)	26 (7.6)
Use of IgR following initiation of rituximab therapy	201 (4.5)
Cancer (n = 3478)	156 (4.5)
Rheumatologic disorder (n = 1241)	31 (2.5)
CVID (n = 57)	13 (2.3)
Hematologic disorder (n = 340)	33 (9.7)

Abbreviations: CVID, common variable immunodeficiency; IgG, immunoglobulin G; IgR, immunoglobulin replacement.

^a^ Hypogammaglobulinemia was defined as a serum IgG level below 600 mg/dL and further stratified into mild, 400 to 599 mg/dL; moderate, 200 to 399 mg/dL; and severe, 0 to 199 mg/dL (to convert to grams per liter, multiply by 0.01).

### Immunologic Evaluation for Hypogammaglobulinemia

Of the patients who received rituximab, 3824 individuals (85.4%) did not have immunoglobulin levels checked in the 12 months before initiation of rituximab therapy. Patient demographics (age, sex, race/ethnicity) were not associated with whether they had their immunoglobulin levels checked. Of those who had immunoglobulin levels determined, 313 patients (47.8%) had mild to severe hypogammaglobulinemia (Table 1). Of these patients, the diagnosis of hypogammaglobulinemia was not coded in 56 of 65 patients (86.1%) with moderate hypogammaglobulinemia, 51 of 91 patients (56.0%) with mild hypogammaglobulinemia, and, most remarkably, 102 of 157 patients (65.0%) with severe hypogammaglobulinemia (IgG level <200 mg/dL).

**Figure 1.**
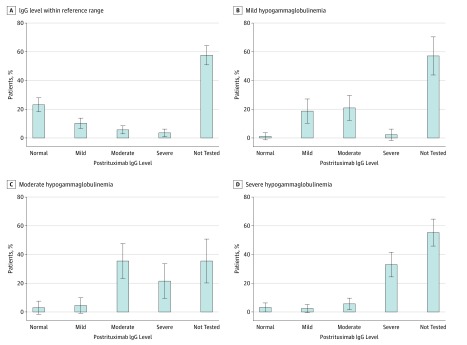
Immunoglobulin G (IgG) Levels 18 Months After Initiation of Rituximab Therapy Levels of IgG after initiation of rituximab therapy in patients with pretherapy status of normogammaglobulinemia (n = 342) (A), mild hypogammaglobulinemia (n = 91) (B), moderate hypogammaglobulinemia (n = 65) (C), and severe hypogammaglobulinemia (n = 157) (D). Error bars indicate 95% CI.

We further evaluated hypogammaglobulinemia following rituximab initiation, stratified by IgG levels before rituximab therapy. Most patients (3921 [87.5%]) did not have their IgG levels measured in the 18 months after rituximab therapy. Among those who had their IgG levels determined both before and after rituximab use, hypogammaglobulinemia appeared to worsen following rituximab use (Figure 1). Sixty-six of 342 patients (19.3%) with normal IgG levels within the reference range before rituximab therapy developed mild to severe hypogammaglobulinemia after rituximab, 21 of 91 patients (23.1%) with mild hypogammaglobulinemia before rituximab developed moderate to severe hypogammaglobulinemia after rituximab, and 14 of 65 patients (21.5%) with moderate hypogammaglobulinemia before rituximab went on to develop severe hypogammaglobulinemia following rituximab use.

### Infectious Complications

**Figure 2.**
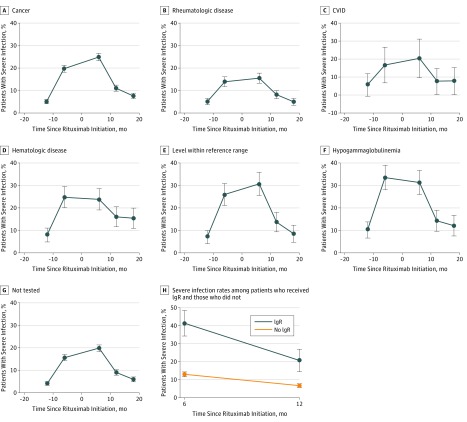
Severe Infection Rates Following Initiation of Rituximab Therapy Infection rates in patients during treatment of cancer (n = 3478) (A), rheumatologic disease (n = 1241) (B), common variable immunodeficiency (CVID) (n = 57), and hematologic disease (n = 340) (D); pretreatment immunoglobulin G (IgG) levels within the reference range (n = 342) (E), showing hypogammaglobulinemia (F), or that were not determined (n = 3824) (G); and comparison of severe infection rates among patients who received immunoglobulin (IgR) replacement therapy (n = 201) and those who did not (n = 4278) in the 6 months following rituximab use (H). Error bars indicate 95% CI.

A total of 1261 patients (28.2%) had severe infections requiring hospitalization in the 18 months following rituximab initiation, most of which occurred within the first 6 months of rituximab use (n = 972). Comparing severe infection rates in the 6 months before and after rituximab initiation, there was a significant increase in the proportion of patients who experienced severe infections following rituximab use in the overall study cohort (from 17.2% to 21.7%; *P* < .001) and among patients with cancer (from 19.1% to 25.1%; *P* < .001) (Figure 2A). Comparison of severe infection rates in the 6 months before and after rituximab treatment did not yield statistically significant differences among patients with rheumatologic disease (from 13.7% to 15.7%; *P* = .17), CVID (from 21.1% to 24.6%; *P* = .11), or hematologic disease (from 24.1% to 23.8%; *P* = .98) (Figure 2B-D). Among patients who had IgG levels within the reference range before rituximab use (Figure 2E), an increase in the rate of severe infections was observed following rituximab use, but the difference did not reach statistical significance (from 26.0% to 30.7%; *P* = .20).

Subgroup analyses of patients who had their IgG level checked before rituximab initiation showed that the severe infection rate was highest among those with hypogammaglobulinemia, with 105 of 313 patients (33.5%) experiencing severe infectious complications before rituximab use (Figure 2F) compared with 597 of 3824 patients (15.6%) who did not have immunoglobulins tested (Figure 2G). Among these patients, the rate of severe infections remained high, with 98 of 313 (31.3%) patients experiencing severe infections following rituximab use (*P* = .61).

### B-Cell Phenotyping

B-cell phenotyping for switched-memory B cells was performed on an insufficient number of patients in the searchable electronic medical record to allow for statistical analysis (n = 37), with longitudinal data (switched-memory B-cell flow cytometry at 3 or more time points) performed on only 5 patients.

### Survival Analysis

In the survival analysis of the overall cohort, increased mortality was associated with increasing age (hazard ratio [HR], 1.02; 95% CI, 1.01-1.02; *P* < .001) and male sex (HR, 1.14; 95% CI, 1.02-1.28; *P* = .02). In the survival analysis of the overall study cohort, increased mortality was associated with the occurrence of serious infectious complications in the 6 months before (HR, 3.14; 95% CI, 2.77-3.55; *P* < .001) and after (HR, 4.97; 95% CI, 4.41-5.60; *P* < .001) the first rituximab infusion (Table 2). The association with serious infectious complications was consistently observed across all disease subgroups (Table 2). The strength of the association between increased mortality and the occurrence of serious infections following rituximab use was greater than the association between increased mortality and the occurrence of serious infections before rituximab use. Subgroup analyses of patients who had hypogammaglobulinemia in the 12 months before the first rituximab infusion and those with hypogammaglobulinemia in the 18 months following the first rituximab use revealed similar patterns of association between mortality risk and infectious complications (Table 2).

**Table 2.** Survival Analysis Examining the Predictors of Mortality Risk Among Patients Who Received Rituximab in the Partners HealthCare System

Covariate	HR (95% CI)	*P* Value
**Primary Analysis Including All Patients (N = 4479)**		
Age	1.02 (1.01-1.02)	<.001
Male sex	1.14 (1.02-1.28)	.02
Serious infections within 180 d		
Before rituximab therapy	3.14 (2.77-3.55)	<.001
After rituximab therapy	4.97 (4.41-5.60)	<.001
Cancer	1.62 (1.31-1.99)	<.001
Rheumatologic disease	0.49 (0.41-0.57)	<.001
Hematologic disorder	0.78 (0.63-0.96)	.02
Common variable immunodeficiency	0.44 (0.21-0.93)	.03
**Subgroup Analysis Including Only Patients With Cancer (n = 3478)**		
Age	1.02 (1.01-1.02)	<.001
Male sex	1.22 (1.09-1.38)	<.001
Serious infections within 180 d		
Before rituximab therapy	2.85 (2.51-3.24)	<.001
After rituximab therapy	5.06 (4.47-5.73)	<.001
**Subgroup Analysis Including Only Patients With Rheumatologic Disease (n = 1241)**		
Age	1.03 (1.02-1.04)	<.001
Male sex	1.42 (1.05-1.93)	.02
Serious infections within 180 d		
Before rituximab therapy	4.06 (2.91-5.68)	<.001
After rituximab therapy	7.06 (5.07-9.84)	<.001
**Subgroup Analysis Including Only Patients With a Hematologic Disorder (n = 340)**		
Age	1.03 (1.02-1.05)	<.001
Male sex	1.02 (0.68-1.54)	.92
Serious infections within 180 d		
Before rituximab therapy	2.43 (1.57-3.78)	<.001
After rituximab therapy	5.60 (3.58-8.77)	<.001
**Subgroup Analysis Including Only Patients With CVID (n = 57)**		
Age	1.01 (0.97-1.06)	.54
Male sex	2.59 (0.59-11.2)	.21
Serious infections within 180 d		
Before rituximab therapy	7.73 (1.31-45.6)	.02
After rituximab therapy	16.0 (1.96-130.0)	.01
**Subgroup Analysis Including Only Patients Who Had Hypogammaglobulinemia Before Rituximab Therapy (n = 313)**		
Age	1.00 (0.99-1.01)	.79
Male sex	1.09 (0.75-1.57)	.65
Serious infections within 180 d		
Before rituximab therapy	4.54 (3.04-6.80)	<.001
After rituximab therapy	3.27 (2.24-4.78)	<.001
Cancer	1.45 (0.75-2.79)	.27
Rheumatologic disease	0.52 (0.34-0.78)	.002
Hematologic disorder	0.53 (0.30-0.93)	.03
Common variable immunodeficiency	0.80 (0.22-2.96)	.74
**Subgroup Analysis Including Only Patients Who Had Hypogammaglobulinemia After Rituximab Therapy (n = 363)**		
Age	1.00 (0.99-1.02)	.94
Male sex	1.08 (0.73-1.61)	.70
Serious infections within 180 d		
Before rituximab therapy	3.23 (2.16-4.82)	<.001
After rituximab therapy	7.20 (4.64-11.1)	<.001
Cancer	1.76 (0.73-4.26)	.21
Rheumatologic disease	0.63 (0.38-1.05)	.07
Hematologic disorder	0.61 (0.92-1.26)	.18
Common variable immunodeficiency	0.00 (0.00-0.00)	<.001

Abbreviations: CVID, common variable immunodeficiency; HR, hazard ratio.

### IgR Treatment Effects

A total of 201 patients (4.5%) received IgR following rituximab therapy. Compared with patients who were not treated with IgR following rituximab, those who received IgR were more likely to have serious infectious complications both 6 months before (32.3% vs 16.5%; *P* < .001) and after (41.4% vs 13.0%; *P* < .001) the first rituximab infusion (Figure 2H). The trend persisted 12 months following the first rituximab infusion (20.7% vs 6.7%; *P* < .001). Application of Cox regression analysis to examine the association between IgR treatment and infectious complications showed a positive association between IgR treatment dose and the occurrence of serious infectious complications following rituximab use (HR, 1.03; 1.02-1.04; *P* < .001). An elevated risk of infectious complications after rituximab therapy was also observed among men, patients with serious infections 6 months before rituximab use, and patients with cancer (Table 3). In the subgroup analysis that included only patients who received IgR treatment, higher cumulative IgR treatment dose was associated with a reduced risk of serious infectious complications (HR, 0.98; 95% CI, 0.96-0.99; *P* = .002).

**Table 3.** Cox Regression Analysis Examining the Association Between IgR and the Occurrence of Serious Infectious Complications Among Patients Who Received Rituximab

Covariate	HR (95% CI)	*P* Value
**Primary Analysis Including All Patients (n = 4479)**		
Age	1.00 (0.99-1.00)	.29
Male sex	1.17 (1.03-1.31)	.01
Serious infections within 180 d before rituximab therapy	4.77 (4.19-5.42)	<.001
IgR following rituximab use, g	1.03 (1.02-1.04)	<.001
Cancer	2.06 (1.67-2.55)	<.001
Rheumatologic disease	0.73 (0.63-0.85)	<.001
Hematologic disorder	1.00 (0.82-1.21)	.98
Common variable immunodeficiency	1.16 (0.78-1.74)	.47
**Subgroup Analysis Including Only Patients Who Received IgR (n = 201)**		
Age	1.00 (0.99-1.01)	.42
Male sex	1.13 (0.81-1.57)	.46
Serious infections within 180 d before rituximab therapy	0.97 (0.66-1.42)	.88
IgR following rituximab use, g	0.98 (0.96-0.99)	.002
Cancer	0.99 (0.64-1.53)	.97
Rheumatologic disease	1.04 (0.58-1.86)	.90
Hematologic disorder	1.22 (0.75-1.98)	.42
Common variable immunodeficiency	0.59 (0.34-1.03)	.06

Abbreviations: HR, hazard ratio; IgR, immunoglobulin replacement.

## Discussion

Rituximab is an important treatment option for a number of indications across a wide range of specialties. Despite its widespread use and reports of prolonged, symptomatic hypogammaglobulinemia following rituximab therapy, there have not been guidelines established for clinical monitoring of immune factors. In this large cohort, we found that most patients (85.4%) did not have immunoglobulin levels checked in the year before the initiation of rituximab. Even in patients with moderate to severe hypogammaglobulinemia, most did not have a diagnosis of hypogammaglobulinemia in their medical record, suggesting that increased awareness is needed regarding the importance of immune evaluation for clinical outcomes, including infection. In our analysis, patients with hypogammaglobulinemia before rituximab administration often went on to develop more severe hypogammaglobulinemia following rituximab use, indicating that routine screening may help to identify patients at increased risk for developing more pronounced hypogammaglobulinemia, which may predispose them to serious infectious complications.

When stratified over time, there was a statistically significant increase in severe infections following rituximab administration beyond the predicted time frame of B-cell depletion based on early studies.^7^ We found that patients who were treated with IgR had a greater rate of serious infectious complications both before and after the first rituximab infusion. This finding suggests that these patients may have had preceding immune dysregulation or dysfunction, including conditions such as CVID, which predisposed these patients to clinically significant infections and prompted IgR therapy. Among patients who received IgR following rituximab use, there was a dose-response relationship between IgR and infection, where higher cumulative doses of IgR were associated with a decreased risk of serious infectious complications. This finding suggests that prompt recognition of symptomatic hypogammaglobulinemia and initiation of IgR may reduce infectious complications for a subset of patients. Conversely, delay in the recognition/diagnosis of hypogammaglobulinemia, as well as in the initiation of IgR for patients with symptomatic hypogammaglobulinemia, may result in an increased number of infections, leading to adverse effects on patient quality of life, morbidity and mortality, and health care–related costs.

In addition to monitoring immunoglobulin levels and infections, polysaccharide and protein vaccine responses may help to identify high-risk patients who may benefit from IgR.^28^ The survival analysis identified several covariates associated with increased mortality in the overall cohort, including older age, male sex, severe infections both before and after rituximab therapy, and cancer.

In our study population, there was an insufficient number of patients with B-cell phenotyping for statistically significant analysis. While memory B-cell depletion has been correlated with clinical response,^27^ it has also been suggested to be a tool for prediction of cell recovery.^8^ As such, we recommend considering determining B-cell flow in addition to immunoglobulin levels.

### Strengths and Limitations

To our knowledge, this is the largest review evaluating hypogammaglobulinemia and rituximab. The number of patients assessed in these analyses allows for increased statistical power, with the possibility of increasing applicability. However, there are several limitations to our study, including that it is a retrospective analysis, subject to selection and misclassification bias. This limitation includes sampling bias, in that differences may exist in patients who had immunoglobulin levels checked regarding the severity of clinical presentation and whether the severity prompted clinical evaluation. In addition, the strict inclusion criterion of an adequate number of follow-up visits before and after rituximab administration, which was used to avoid confounding from prior rituximab exposure from outside referral hospitals, excluded a large number of patients and may limit the applicability of these findings to a broader population. In applying these strict inclusion criteria, the study may be biased toward patients who are more likely to seek care or may have other meaningful underlying differences in their clinical presentation.

The heterogeneity of treatment indications and dosing regimens may mean that these results may not be applicable for all subgroups, and different indications may have intrinsic differences in biological pathways and physiology. For example, analysis of patients with antineutrophil cytoplasmic antibody–associated vasculitis found that serious infections were rare, occurring at a rate of 0.85 per 10 patient-years.^29^ In our analysis, we substratified patients by indication where possible.

Given that this was a retrospective analysis, missing data are also a limitation. Most of our study cohort did not have IgG levels tested before and following rituximab use; we were therefore unable to assess the true prevalence of hypogammaglobulinemia. We analyzed patients at 2 tertiary referral centers, which may limit the generalizability of these results to other settings. In addition, there are a number of confounders that could mask or modulate the overall results, such as comorbid conditions; concurrent chemotherapy or other medications/immune modulators, including corticosteroids; neutropenia; number of cycles of rituximab; or cumulative dose. Although many of these risk factors have been suggested based on studies to date,^30^ the heterogeneity of patients receiving rituximab has limited definitive conclusions, and ongoing studies are needed. For example, neutropenia has been described as developing after rituximab therapy, with possible mechanisms including impaired B-cell recovery and neutrophil kinetics.^20,21^ Patients with recurrent infections after rituximab use may have baseline subclinical immune deficiency or dysfunction, including unrecognized CVID, which is unmasked or exacerbated by rituximab. Further studies are needed to identify risk factors for immune dysfunction following rituximab, and prospective or controlled trials are needed to assess whether screening and early intervention affects outcomes.

## Conclusions

Overall, our data suggest an increased risk of infections and mortality associated with hypogammaglobulinemia after rituximab therapy and highlight the importance of monitoring patients with hypogammaglobulinemia, with immunoglobulin levels determined before and after rituximab therapy. As the use of rituximab continues to increase, it is important for clinicians to be aware of hypogammaglobulinemia associated with the drug. Prior to initiation of rituximab, we recommend routinely checking immunoglobulin levels and baseline B-cell numbers to evaluate for underlying immunodeficiency. If hypogammaglobulinemia is uncovered, we recommend close monitoring for clinical infections and monitoring of laboratory values, with consideration of referral to a clinical immunologist for further evaluation. After completion of rituximab therapy, we recommend periodic laboratory monitoring to identify patients with persistent immune dysfunction who may benefit from IgR.

## Notes

Supplement. **eFigure.** Flow Diagram Illustrating Cohort Inclusion Criteria **eTable.** Distribution of Follow-up Period
