# Supplementary material for: Association of Immunoglobulin Levels, Infectious Risk, and Mortality With Rituximab and Hypogammaglobulinemia
Source: JAMA Netw Open. 2018 Nov 2;1(7):e184169. doi: 10.1001/jamanetworkopen.2018.4169 (PMC6324375; doi:10.1001/jamanetworkopen.2018.4169)
Supplement: Supplement. — eFigure. Flow Diagram Illustrating Cohort Inclusion Criteria eTable. Distribution of Follow-up Period [file jamanetwopen-1-e184169-s001.pdf]

Supplementary Online Content

Barmettler S, Ong M-S, Farmer JR, Choi H, Walter J. Association of immunoglobulin levels, infectious risk, and mortality with rituximab and hypogammaglobulinemia. *JAMA Netw Open*. 2018;1(7):e184169. doi:10.1001/jamanetworkopen.2018.4169

eFigure. Flow Diagram Illustrating Cohort Inclusion Criteria

eTable. Distribution of Follow-up Period

This supplementary material has been provided by the authors to give readers additional information about their work.

© 2018 Barmettler S et al. *JAMA Network Open.*

eFigure. Flow Diagram Illustrating Cohort Inclusion Criteria

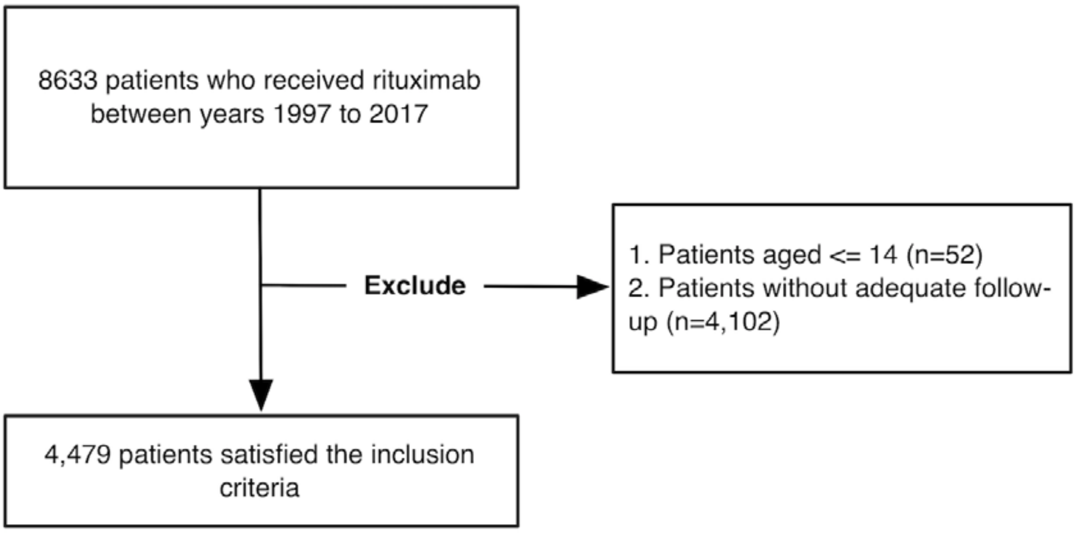

© 2018 Barmettler S et al. *JAMA Network Open.*

eTable 1. Distribution of Follow-up Period

Months since rituximab initiation	Quartile 1	Q2 (Median)	Q3 (Mean)	Q4	Min	Max
-12	4	8	11.8	15	1	181
-6	2	5	8.4	10	1	183
6	8	16	20.5	28	1	182
12	4	7	11.2	14	1	182
18	3	6	9.7	12	1	184

© 2018 Barmettler S et al. *JAMA Network Open.*
